# Supplementary figures and images for: Coronary artery disease classification using ConvMixer based classifier from CT angiography images (part 2 of 2)
Source: PeerJ Comput Sci. 2025 Mar 27;11:e2771. doi: 10.7717/peerj-cs.2771 (PMC12190484; doi:10.7717/peerj-cs.2771)

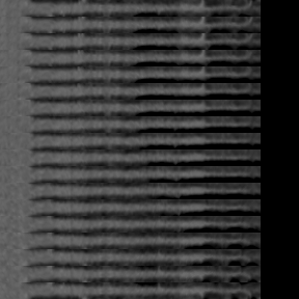

Supplement: Supplemental Information 1 [file peerj-cs-11-2771-s001.zip › sample dataset/Test_images/Test_images/Negative/Negative_Coronary_068_CX_Secondary6_1.png]

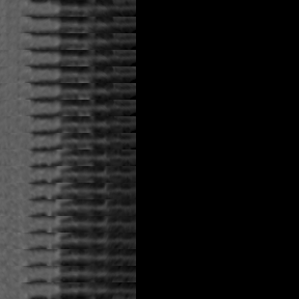

Supplement: Supplemental Information 1 [file peerj-cs-11-2771-s001.zip › sample dataset/Test_images/Test_images/Negative/Negative_Coronary_068_LAD_Secondary1_1.png]

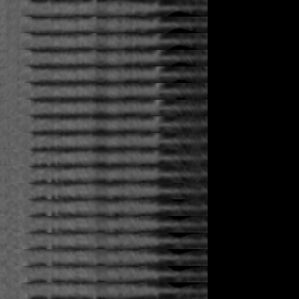

Supplement: Supplemental Information 1 [file peerj-cs-11-2771-s001.zip › sample dataset/Test_images/Test_images/Negative/Negative_Coronary_068_LAD_Secondary2_1.png]

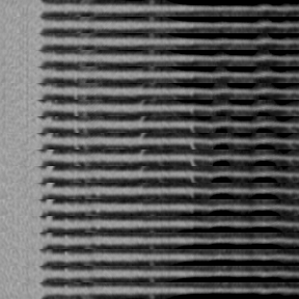

Supplement: Supplemental Information 1 [file peerj-cs-11-2771-s001.zip › sample dataset/Test_images/Test_images/Positive/Positive_Coronary_003_CX1_8.png]

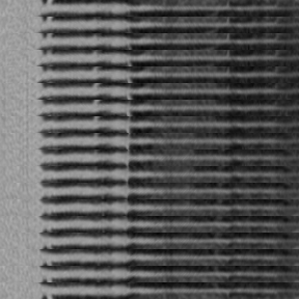

Supplement: Supplemental Information 1 [file peerj-cs-11-2771-s001.zip › sample dataset/Test_images/Test_images/Positive/Positive_Coronary_003_LAD1_5.png]

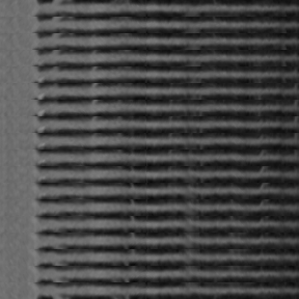

Supplement: Supplemental Information 1 [file peerj-cs-11-2771-s001.zip › sample dataset/Test_images/Test_images/Positive/Positive_Coronary_012_LAD1_7.png]

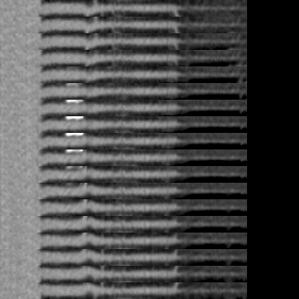

Supplement: Supplemental Information 1 [file peerj-cs-11-2771-s001.zip › sample dataset/Test_images/Test_images/Positive/Positive_Coronary_018_CX1_3.png]

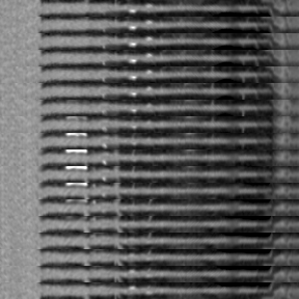

Supplement: Supplemental Information 1 [file peerj-cs-11-2771-s001.zip › sample dataset/Test_images/Test_images/Positive/Positive_Coronary_018_LAD1_5.png]

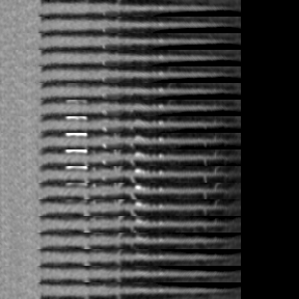

Supplement: Supplemental Information 1 [file peerj-cs-11-2771-s001.zip › sample dataset/Test_images/Test_images/Positive/Positive_Coronary_018_LAD_Secondary2_4.png]

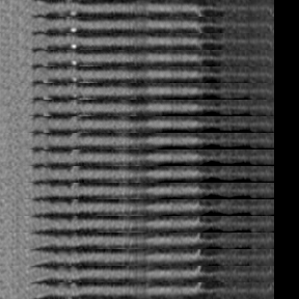

Supplement: Supplemental Information 1 [file peerj-cs-11-2771-s001.zip › sample dataset/Test_images/Test_images/Positive/Positive_Coronary_020_CX_Secondary3_7.png]

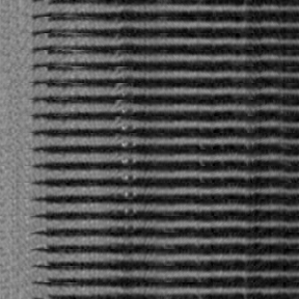

Supplement: Supplemental Information 1 [file peerj-cs-11-2771-s001.zip › sample dataset/Test_images/Test_images/Positive/Positive_Coronary_020_LAD1_5.png]

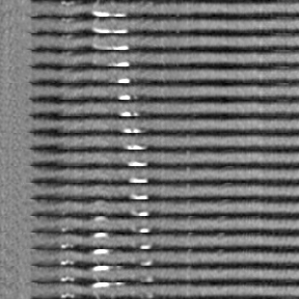

Supplement: Supplemental Information 1 [file peerj-cs-11-2771-s001.zip › sample dataset/Test_images/Test_images/Positive/Positive_Coronary_020_RCA1_5.png]

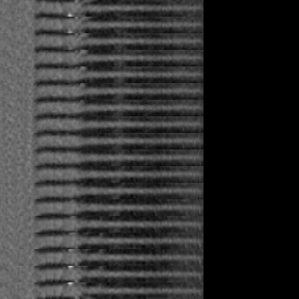

Supplement: Supplemental Information 1 [file peerj-cs-11-2771-s001.zip › sample dataset/Test_images/Test_images/Positive/Positive_Coronary_026_LAD1_7.png]

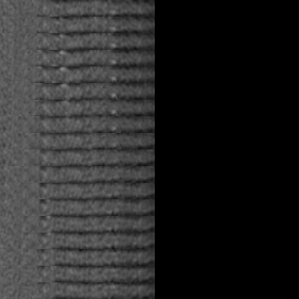

Supplement: Supplemental Information 1 [file peerj-cs-11-2771-s001.zip › sample dataset/Test_images/Test_images/Positive/Positive_Coronary_049_CX1_7.png]

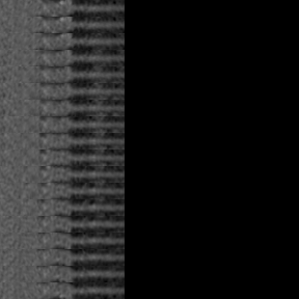

Supplement: Supplemental Information 1 [file peerj-cs-11-2771-s001.zip › sample dataset/Test_images/Test_images/Positive/Positive_Coronary_049_CX_Secondary1_4.png]

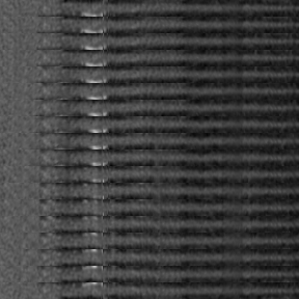

Supplement: Supplemental Information 1 [file peerj-cs-11-2771-s001.zip › sample dataset/Test_images/Test_images/Positive/Positive_Coronary_049_LAD1_8.png]

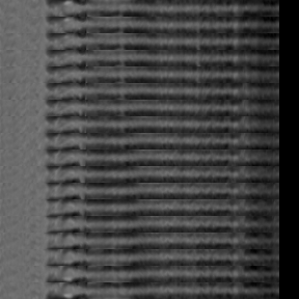

Supplement: Supplemental Information 1 [file peerj-cs-11-2771-s001.zip › sample dataset/Test_images/Test_images/Positive/Positive_Coronary_054_CX_Secondary1_7.png]

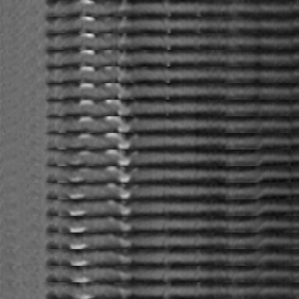

Supplement: Supplemental Information 1 [file peerj-cs-11-2771-s001.zip › sample dataset/Test_images/Test_images/Positive/Positive_Coronary_054_LAD1_1.png]

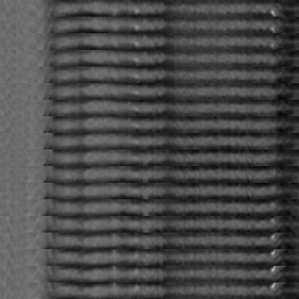

Supplement: Supplemental Information 1 [file peerj-cs-11-2771-s001.zip › sample dataset/Test_images/Test_images/Positive/Positive_Coronary_054_RCA1_3.png]

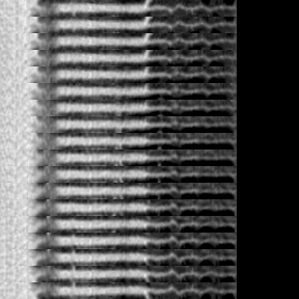

Supplement: Supplemental Information 1 [file peerj-cs-11-2771-s001.zip › sample dataset/Test_images/Test_images/Positive/Positive_Coronary_074_CX1_1.png]

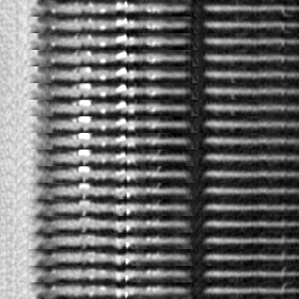

Supplement: Supplemental Information 1 [file peerj-cs-11-2771-s001.zip › sample dataset/Test_images/Test_images/Positive/Positive_Coronary_074_LAD1_2.png]

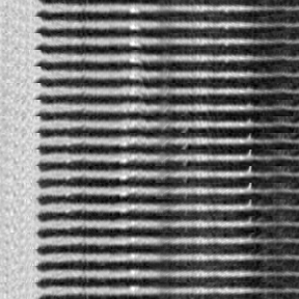

Supplement: Supplemental Information 1 [file peerj-cs-11-2771-s001.zip › sample dataset/Test_images/Test_images/Positive/Positive_Coronary_074_RCA_Secondary1_2.png]

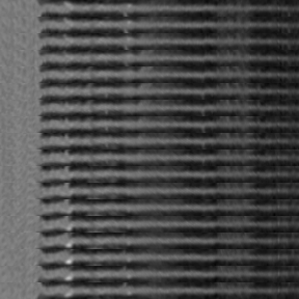

Supplement: Supplemental Information 1 [file peerj-cs-11-2771-s001.zip › sample dataset/Test_images/Test_images/Positive/Positive_Coronary_078_LAD1_8.png]

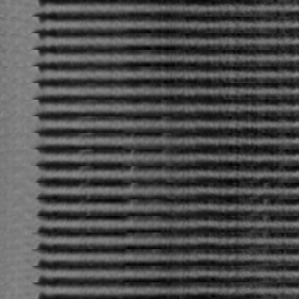

Supplement: Supplemental Information 1 [file peerj-cs-11-2771-s001.zip › sample dataset/Test_images/Test_images/Positive/Positive_Coronary_078_RCA1_3.png]

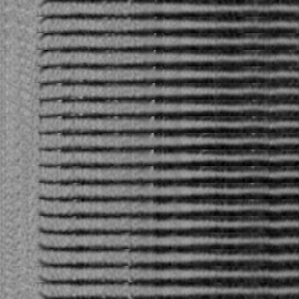

Supplement: Supplemental Information 1 [file peerj-cs-11-2771-s001.zip › sample dataset/Test_images/Test_images/Positive/Positive_Coronary_079_LAD1_3.png]

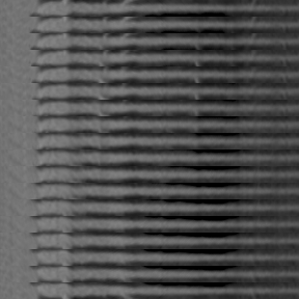

Supplement: Supplemental Information 1 [file peerj-cs-11-2771-s001.zip › sample dataset/Test_images/Test_images/Positive/Positive_Coronary_118_LAD1_1.png]

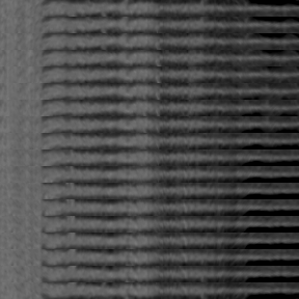

Supplement: Supplemental Information 1 [file peerj-cs-11-2771-s001.zip › sample dataset/Test_images/Test_images/Positive/Positive_Coronary_134_CX1_1.png]

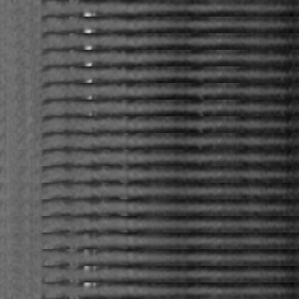

Supplement: Supplemental Information 1 [file peerj-cs-11-2771-s001.zip › sample dataset/Test_images/Test_images/Positive/Positive_Coronary_134_LAD1_5.png]

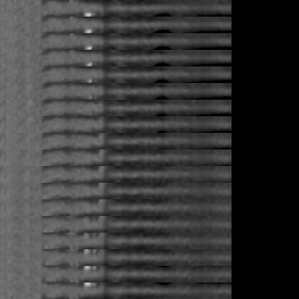

Supplement: Supplemental Information 1 [file peerj-cs-11-2771-s001.zip › sample dataset/Test_images/Test_images/Positive/Positive_Coronary_134_LAD_Secondary2_4.png]

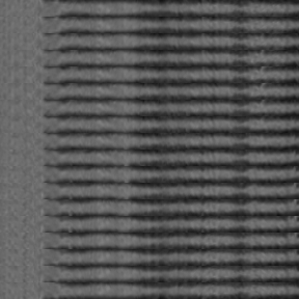

Supplement: Supplemental Information 1 [file peerj-cs-11-2771-s001.zip › sample dataset/Test_images/Test_images/Positive/Positive_Coronary_134_RCA1_1.png]

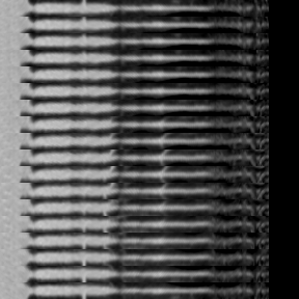

Supplement: Supplemental Information 1 [file peerj-cs-11-2771-s001.zip › sample dataset/Test_images/Test_images/Positive/Positive_Coronary_150_LAD1_8.png]

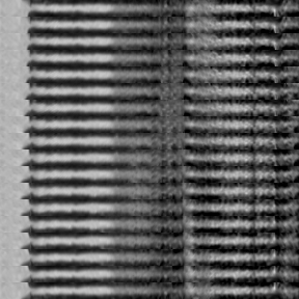

Supplement: Supplemental Information 1 [file peerj-cs-11-2771-s001.zip › sample dataset/Test_images/Test_images/Positive/Positive_Coronary_150_RCA1_8.png]

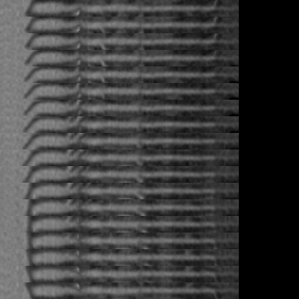

Supplement: Supplemental Information 1 [file peerj-cs-11-2771-s001.zip › sample dataset/Test_images/Test_images/Positive/Positive_Coronary_169_CX1_1.png]

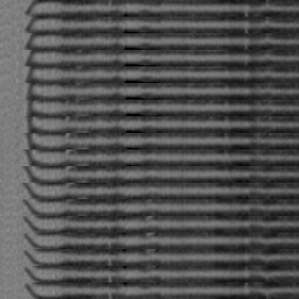

Supplement: Supplemental Information 1 [file peerj-cs-11-2771-s001.zip › sample dataset/Test_images/Test_images/Positive/Positive_Coronary_169_LAD1_3.png]

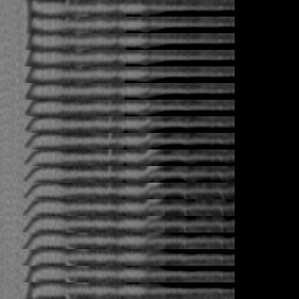

Supplement: Supplemental Information 1 [file peerj-cs-11-2771-s001.zip › sample dataset/Test_images/Test_images/Positive/Positive_Coronary_169_LAD_Secondary1_6.png]

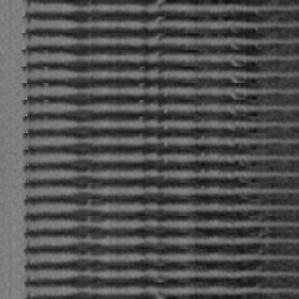

Supplement: Supplemental Information 1 [file peerj-cs-11-2771-s001.zip › sample dataset/Test_images/Test_images/Positive/Positive_Coronary_169_RCA1_8.png]

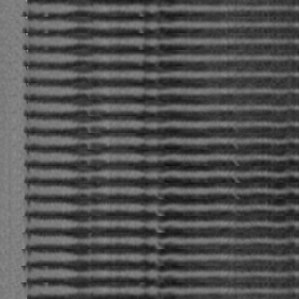

Supplement: Supplemental Information 1 [file peerj-cs-11-2771-s001.zip › sample dataset/Test_images/Test_images/Positive/Positive_Coronary_169_RCA_Secondary1_3.png]

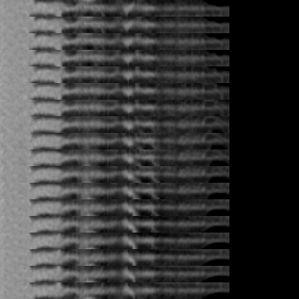

Supplement: Supplemental Information 1 [file peerj-cs-11-2771-s001.zip › sample dataset/Test_images/Test_images/Positive/Positive_Coronary_170_CX1_7.png]

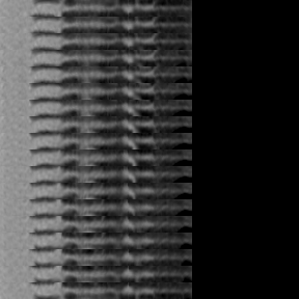

Supplement: Supplemental Information 1 [file peerj-cs-11-2771-s001.zip › sample dataset/Test_images/Test_images/Positive/Positive_Coronary_170_CX_Secondary2_2.png]

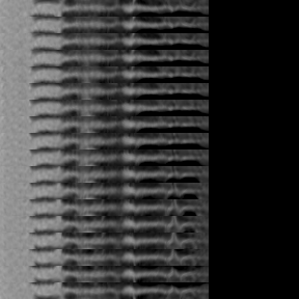

Supplement: Supplemental Information 1 [file peerj-cs-11-2771-s001.zip › sample dataset/Test_images/Test_images/Positive/Positive_Coronary_170_CX_Secondary4_2.png]

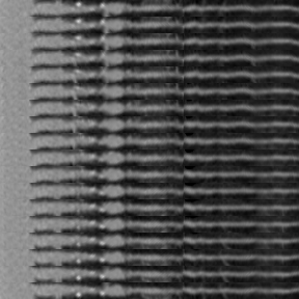

Supplement: Supplemental Information 1 [file peerj-cs-11-2771-s001.zip › sample dataset/Test_images/Test_images/Positive/Positive_Coronary_170_LAD1_2.png]

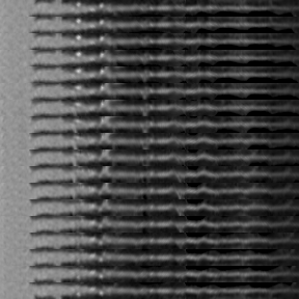

Supplement: Supplemental Information 1 [file peerj-cs-11-2771-s001.zip › sample dataset/Test_images/Test_images/Positive/Positive_Coronary_170_LAD_Secondary1_5.png]

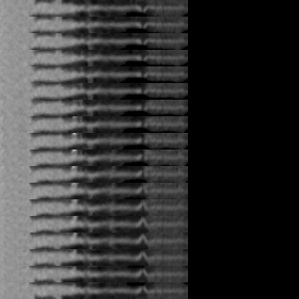

Supplement: Supplemental Information 1 [file peerj-cs-11-2771-s001.zip › sample dataset/Test_images/Test_images/Positive/Positive_Coronary_170_LAD_Secondary2_5.png]

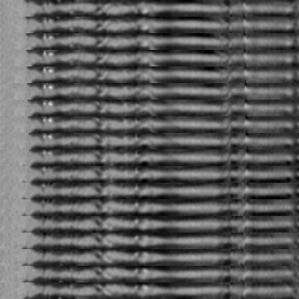

Supplement: Supplemental Information 1 [file peerj-cs-11-2771-s001.zip › sample dataset/Test_images/Test_images/Positive/Positive_Coronary_170_RCA1_3.png]

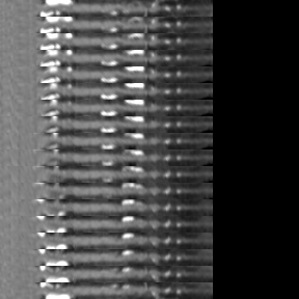

Supplement: Supplemental Information 1 [file peerj-cs-11-2771-s001.zip › sample dataset/Test_images/Test_images/Positive/Positive_Coronary_186_CX1_1.png]

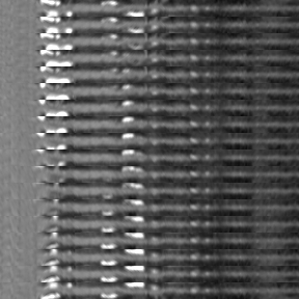

Supplement: Supplemental Information 1 [file peerj-cs-11-2771-s001.zip › sample dataset/Test_images/Test_images/Positive/Positive_Coronary_186_CX_Secondary1_4.png]

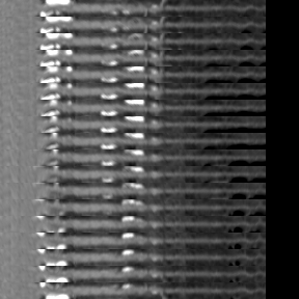

Supplement: Supplemental Information 1 [file peerj-cs-11-2771-s001.zip › sample dataset/Test_images/Test_images/Positive/Positive_Coronary_186_CX_Secondary2_2.png]

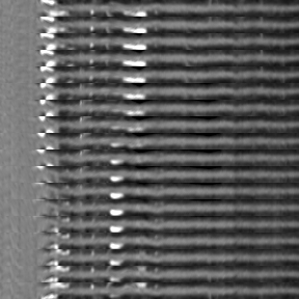

Supplement: Supplemental Information 1 [file peerj-cs-11-2771-s001.zip › sample dataset/Test_images/Test_images/Positive/Positive_Coronary_186_LAD1_5.png]

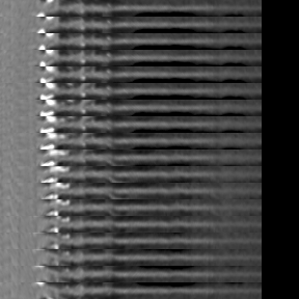

Supplement: Supplemental Information 1 [file peerj-cs-11-2771-s001.zip › sample dataset/Test_images/Test_images/Positive/Positive_Coronary_186_LAD_Secondary1_6.png]

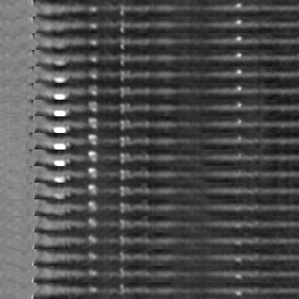

Supplement: Supplemental Information 1 [file peerj-cs-11-2771-s001.zip › sample dataset/Test_images/Test_images/Positive/Positive_Coronary_186_RCA_Secondary4_8.png]

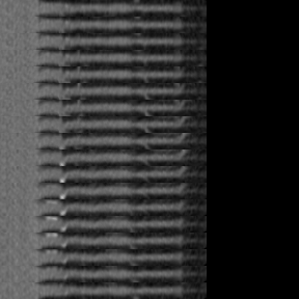

Supplement: Supplemental Information 1 [file peerj-cs-11-2771-s001.zip › sample dataset/Test_images/Test_images/Positive/Positive_Coronary_187_CX_Secondary5_8.png]

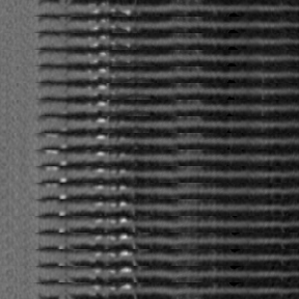

Supplement: Supplemental Information 1 [file peerj-cs-11-2771-s001.zip › sample dataset/Test_images/Test_images/Positive/Positive_Coronary_187_LAD1_4.png]

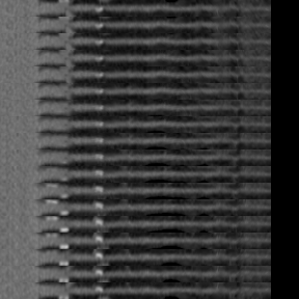

Supplement: Supplemental Information 1 [file peerj-cs-11-2771-s001.zip › sample dataset/Test_images/Test_images/Positive/Positive_Coronary_187_LAD_Secondary1_7.png]

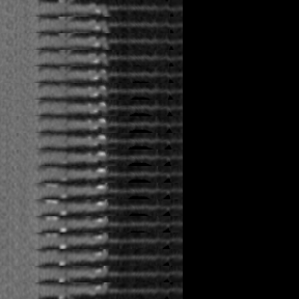

Supplement: Supplemental Information 1 [file peerj-cs-11-2771-s001.zip › sample dataset/Test_images/Test_images/Positive/Positive_Coronary_187_LAD_Secondary4_7.png]

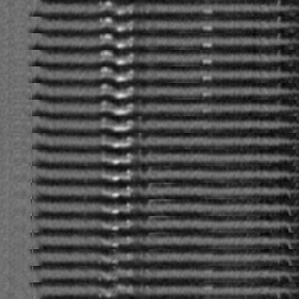

Supplement: Supplemental Information 1 [file peerj-cs-11-2771-s001.zip › sample dataset/Test_images/Test_images/Positive/Positive_Coronary_187_RCA1_2.png]

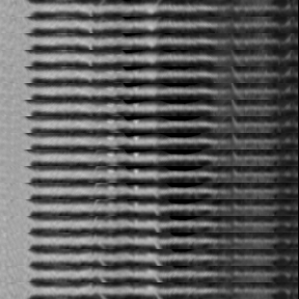

Supplement: Supplemental Information 1 [file peerj-cs-11-2771-s001.zip › sample dataset/Test_images/Test_images/Positive/Positive_Coronary_198_LAD1_6.png]

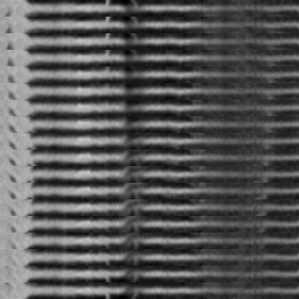

Supplement: Supplemental Information 1 [file peerj-cs-11-2771-s001.zip › sample dataset/Test_images/Test_images/Positive/Positive_Coronary_198_RCA1_4.png]

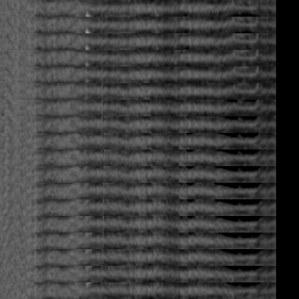

Supplement: Supplemental Information 1 [file peerj-cs-11-2771-s001.zip › sample dataset/Test_images/Test_images/Positive/Positive_Coronary_220_CX_Secondary2_6.png]

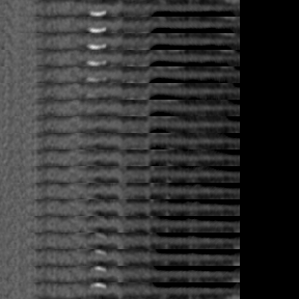

Supplement: Supplemental Information 1 [file peerj-cs-11-2771-s001.zip › sample dataset/Test_images/Test_images/Positive/Positive_Coronary_220_LAD_Secondary1_2.png]

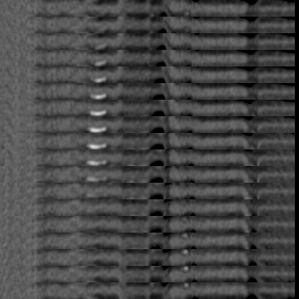

Supplement: Supplemental Information 1 [file peerj-cs-11-2771-s001.zip › sample dataset/Test_images/Test_images/Positive/Positive_Coronary_220_LAD_Secondary2_8.png]

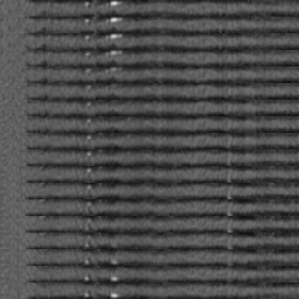

Supplement: Supplemental Information 1 [file peerj-cs-11-2771-s001.zip › sample dataset/Test_images/Test_images/Positive/Positive_Coronary_220_RCA_Secondary2_6.png]

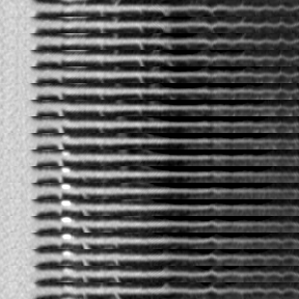

Supplement: Supplemental Information 1 [file peerj-cs-11-2771-s001.zip › sample dataset/Test_images/Test_images/Positive/Positive_Coronary_223_LAD1_5.png]

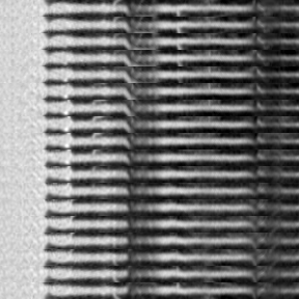

Supplement: Supplemental Information 1 [file peerj-cs-11-2771-s001.zip › sample dataset/Test_images/Test_images/Positive/Positive_Coronary_228_LAD1_1.png]

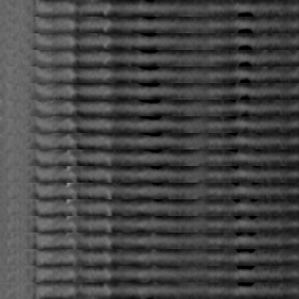

Supplement: Supplemental Information 1 [file peerj-cs-11-2771-s001.zip › sample dataset/Test_images/Test_images/Positive/Positive_Coronary_449_LAD1_8.png]

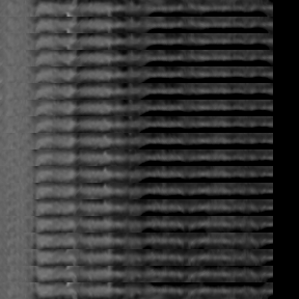

Supplement: Supplemental Information 1 [file peerj-cs-11-2771-s001.zip › sample dataset/Test_images/Test_images/Positive/Positive_Coronary_449_LAD_Secondary1_5.png]

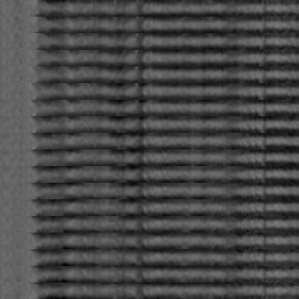

Supplement: Supplemental Information 1 [file peerj-cs-11-2771-s001.zip › sample dataset/Test_images/Test_images/Positive/Positive_Coronary_449_RCA1_3.png]

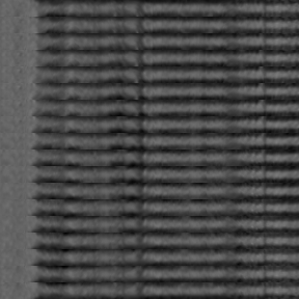

Supplement: Supplemental Information 1 [file peerj-cs-11-2771-s001.zip › sample dataset/Test_images/Test_images/Positive/Positive_Coronary_449_RCA_Secondary2_3.png]

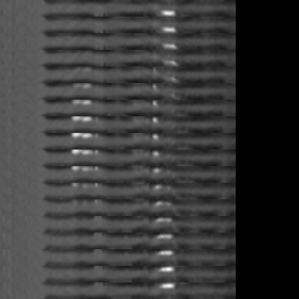

Supplement: Supplemental Information 1 [file peerj-cs-11-2771-s001.zip › sample dataset/Test_images/Test_images/Positive/Positive_Coronary_452_CX1_6.png]

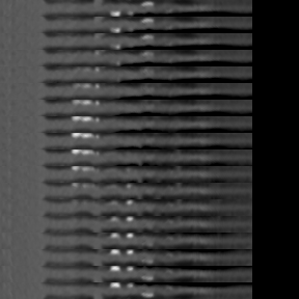

Supplement: Supplemental Information 1 [file peerj-cs-11-2771-s001.zip › sample dataset/Test_images/Test_images/Positive/Positive_Coronary_452_LAD1_6.png]

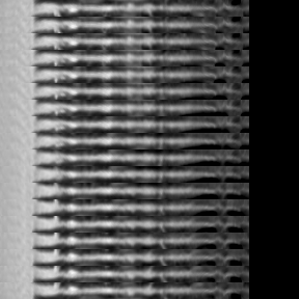

Supplement: Supplemental Information 1 [file peerj-cs-11-2771-s001.zip › sample dataset/Test_images/Test_images/Positive/Positive_Coronary_454_CX1_3.png]

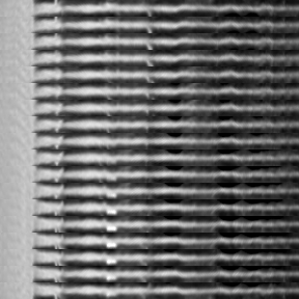

Supplement: Supplemental Information 1 [file peerj-cs-11-2771-s001.zip › sample dataset/Test_images/Test_images/Positive/Positive_Coronary_454_LAD1_4.png]

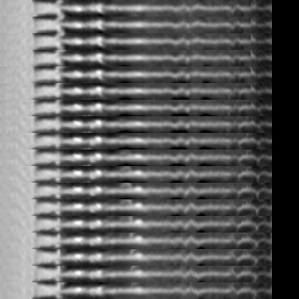

Supplement: Supplemental Information 1 [file peerj-cs-11-2771-s001.zip › sample dataset/Test_images/Test_images/Positive/Positive_Coronary_454_LAD_Secondary1_8.png]

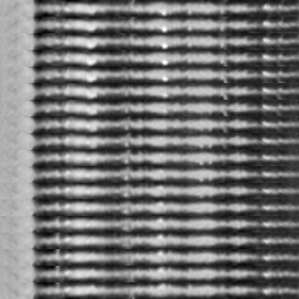

Supplement: Supplemental Information 1 [file peerj-cs-11-2771-s001.zip › sample dataset/Test_images/Test_images/Positive/Positive_Coronary_454_RCA1_8.png]

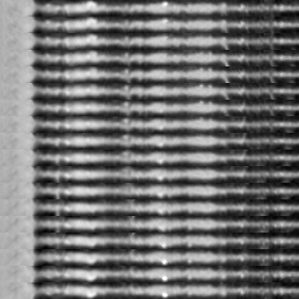

Supplement: Supplemental Information 1 [file peerj-cs-11-2771-s001.zip › sample dataset/Test_images/Test_images/Positive/Positive_Coronary_454_RCA_Secondary1_4.png]

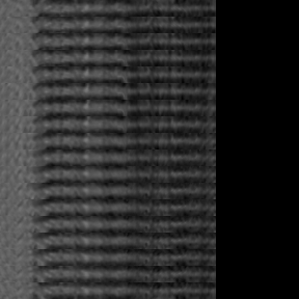

Supplement: Supplemental Information 1 [file peerj-cs-11-2771-s001.zip › sample dataset/Test_images/Test_images/Positive/Positive_Coronary_455_LAD1_3.png]

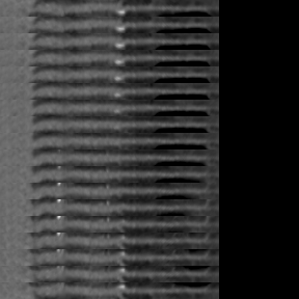

Supplement: Supplemental Information 1 [file peerj-cs-11-2771-s001.zip › sample dataset/Test_images/Test_images/Positive/Positive_Coronary_458_CX_Secondary9_1.png]

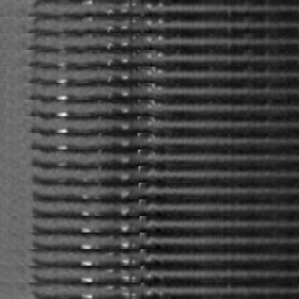

Supplement: Supplemental Information 1 [file peerj-cs-11-2771-s001.zip › sample dataset/Test_images/Test_images/Positive/Positive_Coronary_458_LAD1_2.png]

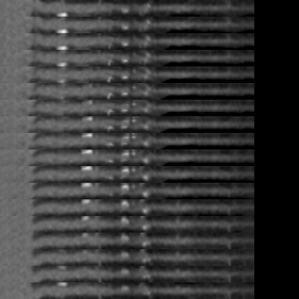

Supplement: Supplemental Information 1 [file peerj-cs-11-2771-s001.zip › sample dataset/Test_images/Test_images/Positive/Positive_Coronary_458_LAD_Secondary4_7.png]

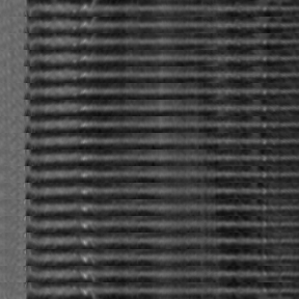

Supplement: Supplemental Information 1 [file peerj-cs-11-2771-s001.zip › sample dataset/Test_images/Test_images/Positive/Positive_Coronary_458_RCA1_6.png]

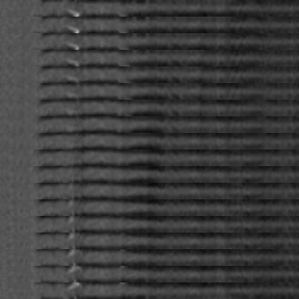

Supplement: Supplemental Information 1 [file peerj-cs-11-2771-s001.zip › sample dataset/Test_images/Test_images/Positive/Positive_Coronary_463_LAD1_1.png]

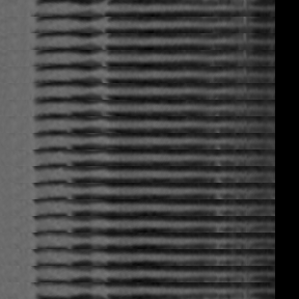

Supplement: Supplemental Information 1 [file peerj-cs-11-2771-s001.zip › sample dataset/Test_images/Test_images/Positive/Positive_Coronary_478_LAD1_1.png]

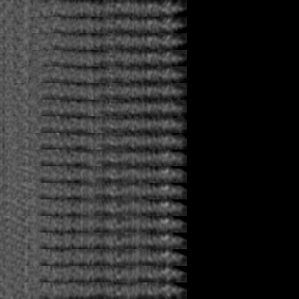

Supplement: Supplemental Information 1 [file peerj-cs-11-2771-s001.zip › sample dataset/Test_images/Test_images/Positive/Positive_Coronary_479_CX1_4.png]

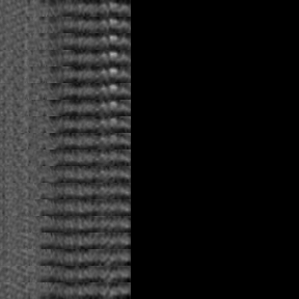

Supplement: Supplemental Information 1 [file peerj-cs-11-2771-s001.zip › sample dataset/Test_images/Test_images/Positive/Positive_Coronary_479_CX_Secondary1_8.png]

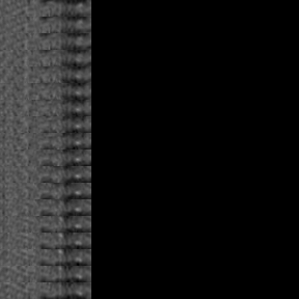

Supplement: Supplemental Information 1 [file peerj-cs-11-2771-s001.zip › sample dataset/Test_images/Test_images/Positive/Positive_Coronary_479_CX_Secondary4_8.png]

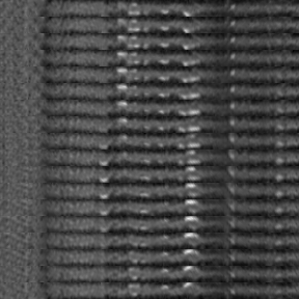

Supplement: Supplemental Information 1 [file peerj-cs-11-2771-s001.zip › sample dataset/Test_images/Test_images/Positive/Positive_Coronary_479_LAD1_5.png]

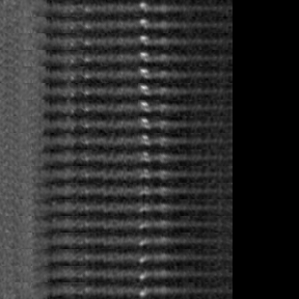

Supplement: Supplemental Information 1 [file peerj-cs-11-2771-s001.zip › sample dataset/Test_images/Test_images/Positive/Positive_Coronary_479_RCA1_2.png]

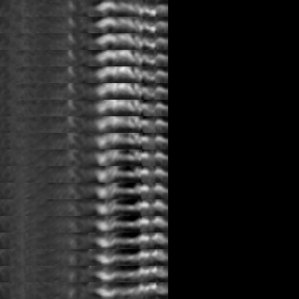

Supplement: Supplemental Information 1 [file peerj-cs-11-2771-s001.zip › sample dataset/Test_images/Test_images/Positive/Positive_Coronary_483_LAD1_1.png]

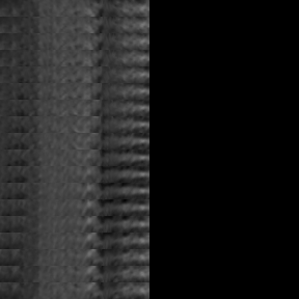

Supplement: Supplemental Information 1 [file peerj-cs-11-2771-s001.zip › sample dataset/Test_images/Test_images/Positive/Positive_Coronary_483_RCA1_2.png]

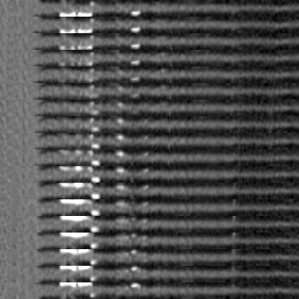

Supplement: Supplemental Information 1 [file peerj-cs-11-2771-s001.zip › sample dataset/Test_images/Test_images/Positive/Positive_Coronary_490_LAD1_2.png]

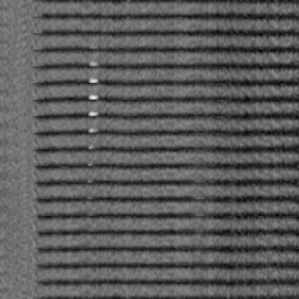

Supplement: Supplemental Information 1 [file peerj-cs-11-2771-s001.zip › sample dataset/Test_images/Test_images/Positive/Positive_Coronary_490_RCA1_1.png]

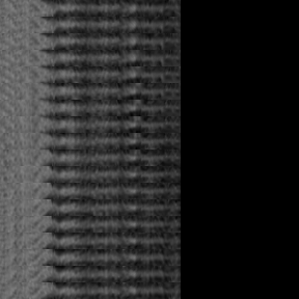

Supplement: Supplemental Information 1 [file peerj-cs-11-2771-s001.zip › sample dataset/Test_images/Test_images/Positive/Positive_Coronary_493_CX1_5.png]

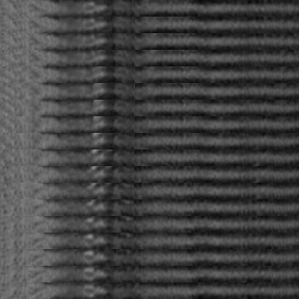

Supplement: Supplemental Information 1 [file peerj-cs-11-2771-s001.zip › sample dataset/Test_images/Test_images/Positive/Positive_Coronary_493_LAD1_6.png]

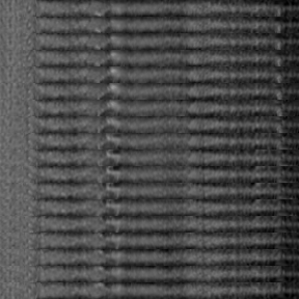

Supplement: Supplemental Information 1 [file peerj-cs-11-2771-s001.zip › sample dataset/Test_images/Test_images/Positive/Positive_Coronary_493_RCA1_3.png]
