# Supplementary figures and images for: Coronary artery disease classification using ConvMixer based classifier from CT angiography images (part 1 of 2)
Source: PeerJ Comput Sci. 2025 Mar 27;11:e2771. doi: 10.7717/peerj-cs.2771 (PMC12190484; doi:10.7717/peerj-cs.2771)

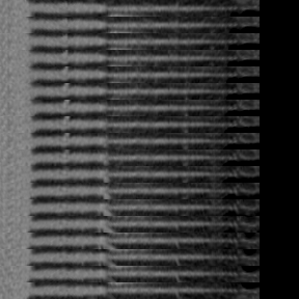

Supplement: Supplemental Information 1 [file peerj-cs-11-2771-s001.zip › sample dataset/Test_images/Test_images/Negative/Negative_Coronary_008_CX1_1.png]

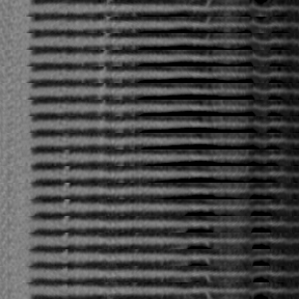

Supplement: Supplemental Information 1 [file peerj-cs-11-2771-s001.zip › sample dataset/Test_images/Test_images/Negative/Negative_Coronary_008_CX_Secondary1_1.png]

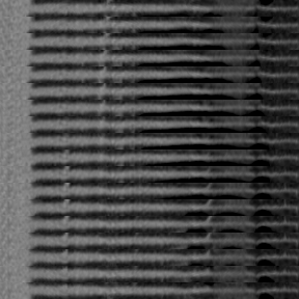

Supplement: Supplemental Information 1 [file peerj-cs-11-2771-s001.zip › sample dataset/Test_images/Test_images/Negative/Negative_Coronary_008_CX_Secondary2_1.png]

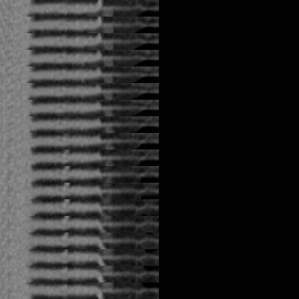

Supplement: Supplemental Information 1 [file peerj-cs-11-2771-s001.zip › sample dataset/Test_images/Test_images/Negative/Negative_Coronary_008_CX_Secondary3_1.png]

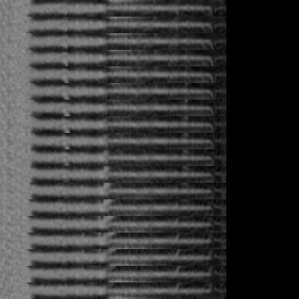

Supplement: Supplemental Information 1 [file peerj-cs-11-2771-s001.zip › sample dataset/Test_images/Test_images/Negative/Negative_Coronary_008_CX_Secondary4_1.png]

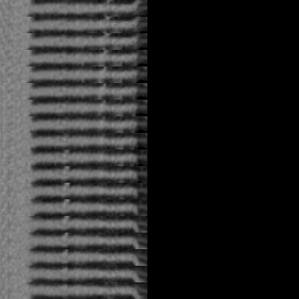

Supplement: Supplemental Information 1 [file peerj-cs-11-2771-s001.zip › sample dataset/Test_images/Test_images/Negative/Negative_Coronary_008_CX_Secondary5_1.png]

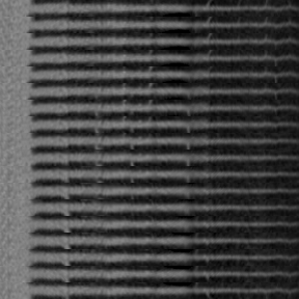

Supplement: Supplemental Information 1 [file peerj-cs-11-2771-s001.zip › sample dataset/Test_images/Test_images/Negative/Negative_Coronary_008_LAD1_1.png]

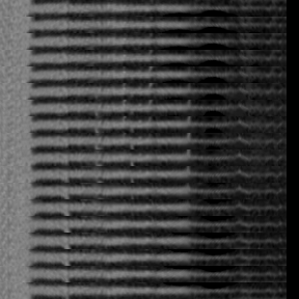

Supplement: Supplemental Information 1 [file peerj-cs-11-2771-s001.zip › sample dataset/Test_images/Test_images/Negative/Negative_Coronary_008_LAD_Secondary1_1.png]

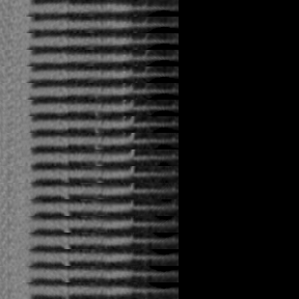

Supplement: Supplemental Information 1 [file peerj-cs-11-2771-s001.zip › sample dataset/Test_images/Test_images/Negative/Negative_Coronary_008_LAD_Secondary2_1.png]

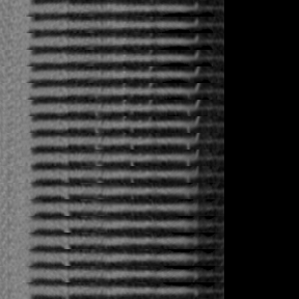

Supplement: Supplemental Information 1 [file peerj-cs-11-2771-s001.zip › sample dataset/Test_images/Test_images/Negative/Negative_Coronary_008_LAD_Secondary3_1.png]

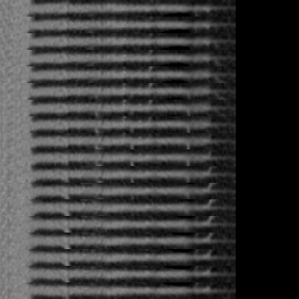

Supplement: Supplemental Information 1 [file peerj-cs-11-2771-s001.zip › sample dataset/Test_images/Test_images/Negative/Negative_Coronary_008_LAD_Secondary4_1.png]

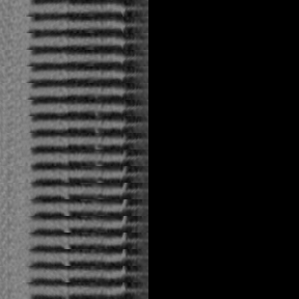

Supplement: Supplemental Information 1 [file peerj-cs-11-2771-s001.zip › sample dataset/Test_images/Test_images/Negative/Negative_Coronary_008_LAD_Secondary5_1.png]

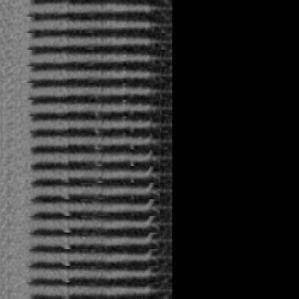

Supplement: Supplemental Information 1 [file peerj-cs-11-2771-s001.zip › sample dataset/Test_images/Test_images/Negative/Negative_Coronary_008_LAD_Secondary6_1.png]

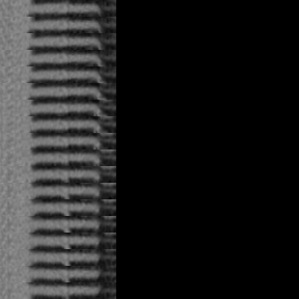

Supplement: Supplemental Information 1 [file peerj-cs-11-2771-s001.zip › sample dataset/Test_images/Test_images/Negative/Negative_Coronary_008_LAD_Secondary7_1.png]

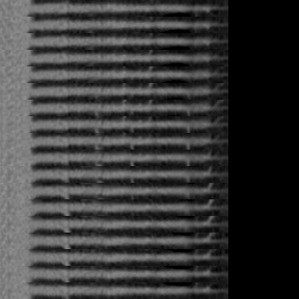

Supplement: Supplemental Information 1 [file peerj-cs-11-2771-s001.zip › sample dataset/Test_images/Test_images/Negative/Negative_Coronary_008_LAD_Secondary8_1.png]

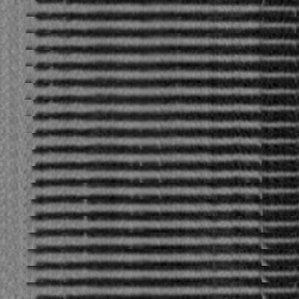

Supplement: Supplemental Information 1 [file peerj-cs-11-2771-s001.zip › sample dataset/Test_images/Test_images/Negative/Negative_Coronary_008_RCA1_1.png]

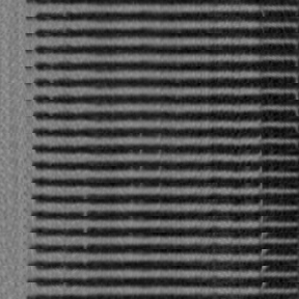

Supplement: Supplemental Information 1 [file peerj-cs-11-2771-s001.zip › sample dataset/Test_images/Test_images/Negative/Negative_Coronary_008_RCA_Secondary10_1.png]

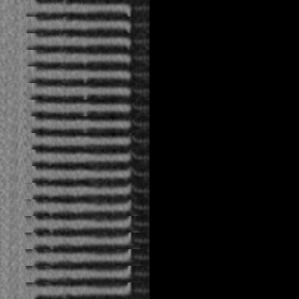

Supplement: Supplemental Information 1 [file peerj-cs-11-2771-s001.zip › sample dataset/Test_images/Test_images/Negative/Negative_Coronary_008_RCA_Secondary11_1.png]

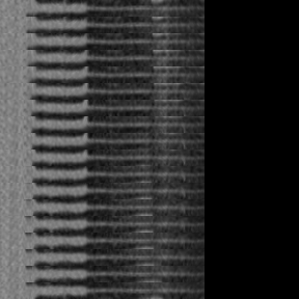

Supplement: Supplemental Information 1 [file peerj-cs-11-2771-s001.zip › sample dataset/Test_images/Test_images/Negative/Negative_Coronary_008_RCA_Secondary12_1.png]

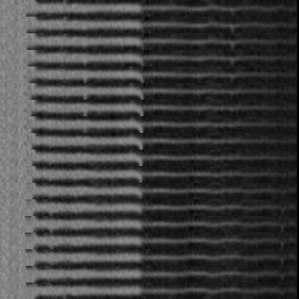

Supplement: Supplemental Information 1 [file peerj-cs-11-2771-s001.zip › sample dataset/Test_images/Test_images/Negative/Negative_Coronary_008_RCA_Secondary1_1.png]

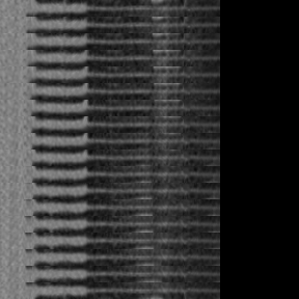

Supplement: Supplemental Information 1 [file peerj-cs-11-2771-s001.zip › sample dataset/Test_images/Test_images/Negative/Negative_Coronary_008_RCA_Secondary2_1.png]

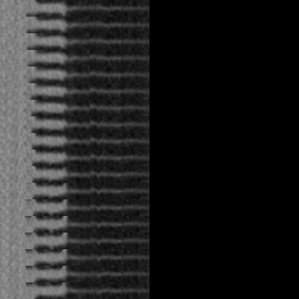

Supplement: Supplemental Information 1 [file peerj-cs-11-2771-s001.zip › sample dataset/Test_images/Test_images/Negative/Negative_Coronary_008_RCA_Secondary3_1.png]

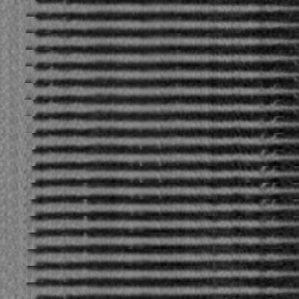

Supplement: Supplemental Information 1 [file peerj-cs-11-2771-s001.zip › sample dataset/Test_images/Test_images/Negative/Negative_Coronary_008_RCA_Secondary4_1.png]

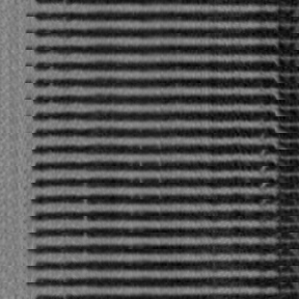

Supplement: Supplemental Information 1 [file peerj-cs-11-2771-s001.zip › sample dataset/Test_images/Test_images/Negative/Negative_Coronary_008_RCA_Secondary5_1.png]

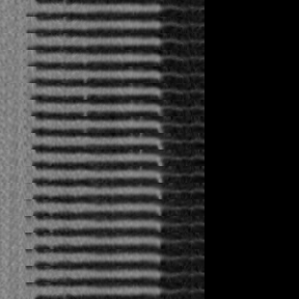

Supplement: Supplemental Information 1 [file peerj-cs-11-2771-s001.zip › sample dataset/Test_images/Test_images/Negative/Negative_Coronary_008_RCA_Secondary6_1.png]

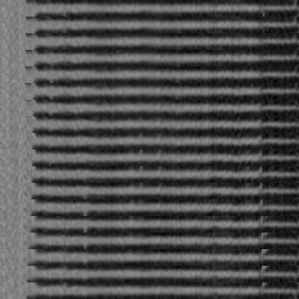

Supplement: Supplemental Information 1 [file peerj-cs-11-2771-s001.zip › sample dataset/Test_images/Test_images/Negative/Negative_Coronary_008_RCA_Secondary7_1.png]

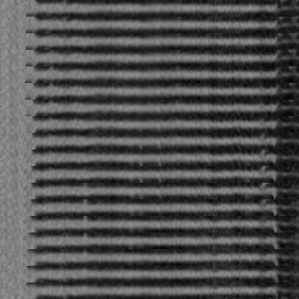

Supplement: Supplemental Information 1 [file peerj-cs-11-2771-s001.zip › sample dataset/Test_images/Test_images/Negative/Negative_Coronary_008_RCA_Secondary8_1.png]

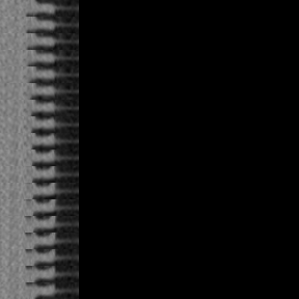

Supplement: Supplemental Information 1 [file peerj-cs-11-2771-s001.zip › sample dataset/Test_images/Test_images/Negative/Negative_Coronary_008_RCA_Secondary9_1.png]

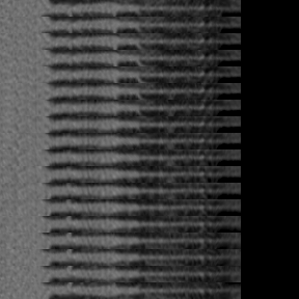

Supplement: Supplemental Information 1 [file peerj-cs-11-2771-s001.zip › sample dataset/Test_images/Test_images/Negative/Negative_Coronary_021_CX1_1.png]

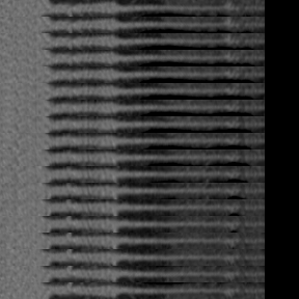

Supplement: Supplemental Information 1 [file peerj-cs-11-2771-s001.zip › sample dataset/Test_images/Test_images/Negative/Negative_Coronary_021_CX_Secondary1_1.png]

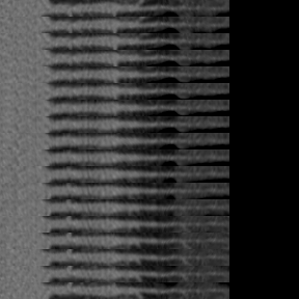

Supplement: Supplemental Information 1 [file peerj-cs-11-2771-s001.zip › sample dataset/Test_images/Test_images/Negative/Negative_Coronary_021_CX_Secondary2_1.png]

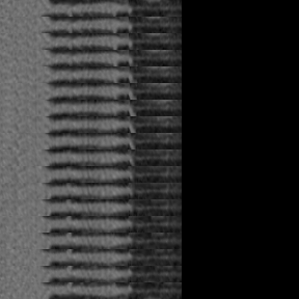

Supplement: Supplemental Information 1 [file peerj-cs-11-2771-s001.zip › sample dataset/Test_images/Test_images/Negative/Negative_Coronary_021_CX_Secondary3_1.png]

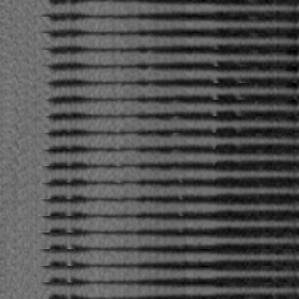

Supplement: Supplemental Information 1 [file peerj-cs-11-2771-s001.zip › sample dataset/Test_images/Test_images/Negative/Negative_Coronary_021_LAD1_1.png]

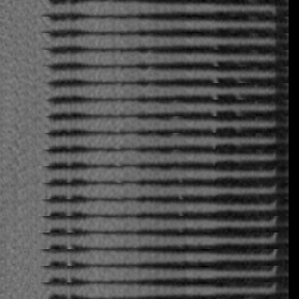

Supplement: Supplemental Information 1 [file peerj-cs-11-2771-s001.zip › sample dataset/Test_images/Test_images/Negative/Negative_Coronary_021_LAD_Secondary10_1.png]

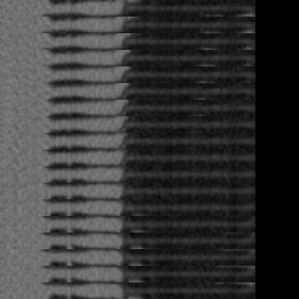

Supplement: Supplemental Information 1 [file peerj-cs-11-2771-s001.zip › sample dataset/Test_images/Test_images/Negative/Negative_Coronary_021_LAD_Secondary1_1.png]

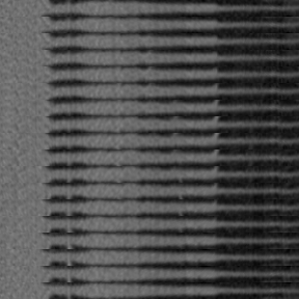

Supplement: Supplemental Information 1 [file peerj-cs-11-2771-s001.zip › sample dataset/Test_images/Test_images/Negative/Negative_Coronary_021_LAD_Secondary2_1.png]

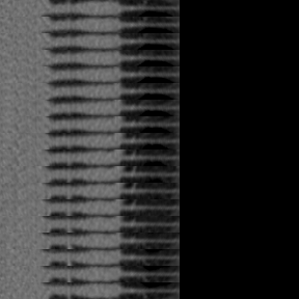

Supplement: Supplemental Information 1 [file peerj-cs-11-2771-s001.zip › sample dataset/Test_images/Test_images/Negative/Negative_Coronary_021_LAD_Secondary3_1.png]

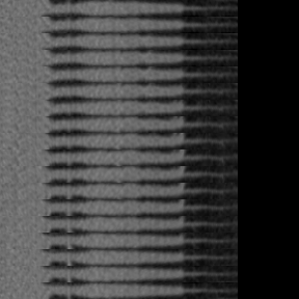

Supplement: Supplemental Information 1 [file peerj-cs-11-2771-s001.zip › sample dataset/Test_images/Test_images/Negative/Negative_Coronary_021_LAD_Secondary4_1.png]

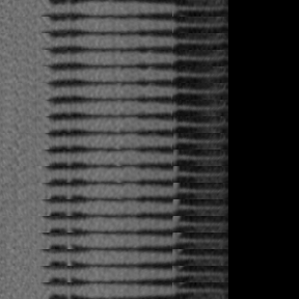

Supplement: Supplemental Information 1 [file peerj-cs-11-2771-s001.zip › sample dataset/Test_images/Test_images/Negative/Negative_Coronary_021_LAD_Secondary5_1.png]

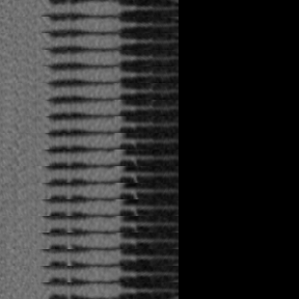

Supplement: Supplemental Information 1 [file peerj-cs-11-2771-s001.zip › sample dataset/Test_images/Test_images/Negative/Negative_Coronary_021_LAD_Secondary6_1.png]

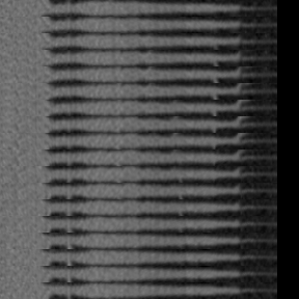

Supplement: Supplemental Information 1 [file peerj-cs-11-2771-s001.zip › sample dataset/Test_images/Test_images/Negative/Negative_Coronary_021_LAD_Secondary7_1.png]

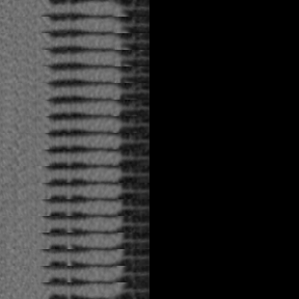

Supplement: Supplemental Information 1 [file peerj-cs-11-2771-s001.zip › sample dataset/Test_images/Test_images/Negative/Negative_Coronary_021_LAD_Secondary8_1.png]

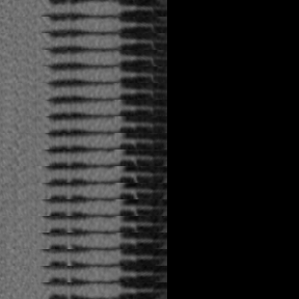

Supplement: Supplemental Information 1 [file peerj-cs-11-2771-s001.zip › sample dataset/Test_images/Test_images/Negative/Negative_Coronary_021_LAD_Secondary9_1.png]

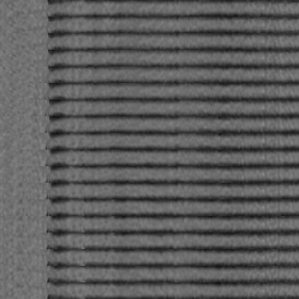

Supplement: Supplemental Information 1 [file peerj-cs-11-2771-s001.zip › sample dataset/Test_images/Test_images/Negative/Negative_Coronary_021_RCA1_1.png]

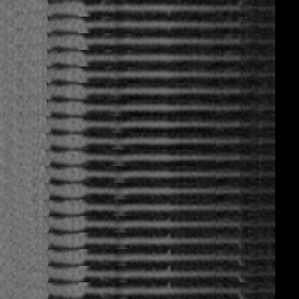

Supplement: Supplemental Information 1 [file peerj-cs-11-2771-s001.zip › sample dataset/Test_images/Test_images/Negative/Negative_Coronary_021_RCA_Secondary10_1.png]

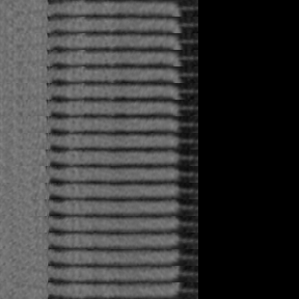

Supplement: Supplemental Information 1 [file peerj-cs-11-2771-s001.zip › sample dataset/Test_images/Test_images/Negative/Negative_Coronary_021_RCA_Secondary11_1.png]

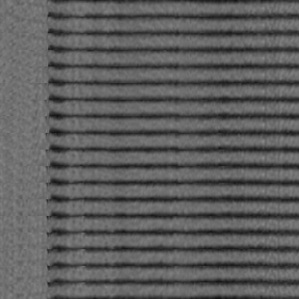

Supplement: Supplemental Information 1 [file peerj-cs-11-2771-s001.zip › sample dataset/Test_images/Test_images/Negative/Negative_Coronary_021_RCA_Secondary12_1.png]

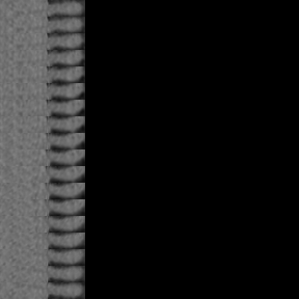

Supplement: Supplemental Information 1 [file peerj-cs-11-2771-s001.zip › sample dataset/Test_images/Test_images/Negative/Negative_Coronary_021_RCA_Secondary15_1.png]

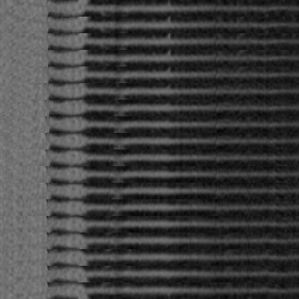

Supplement: Supplemental Information 1 [file peerj-cs-11-2771-s001.zip › sample dataset/Test_images/Test_images/Negative/Negative_Coronary_021_RCA_Secondary1_1.png]

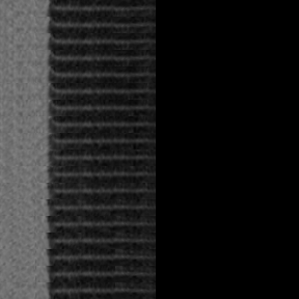

Supplement: Supplemental Information 1 [file peerj-cs-11-2771-s001.zip › sample dataset/Test_images/Test_images/Negative/Negative_Coronary_021_RCA_Secondary3_1.png]

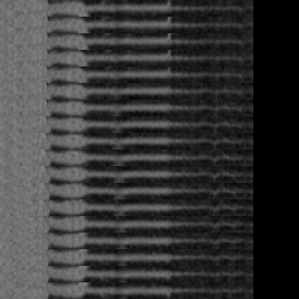

Supplement: Supplemental Information 1 [file peerj-cs-11-2771-s001.zip › sample dataset/Test_images/Test_images/Negative/Negative_Coronary_021_RCA_Secondary4_1.png]

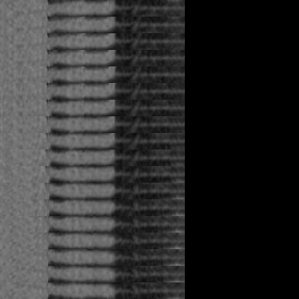

Supplement: Supplemental Information 1 [file peerj-cs-11-2771-s001.zip › sample dataset/Test_images/Test_images/Negative/Negative_Coronary_021_RCA_Secondary5_1.png]

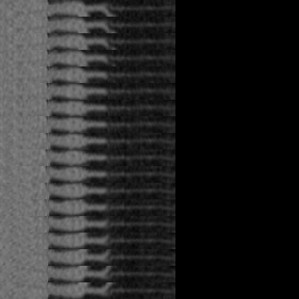

Supplement: Supplemental Information 1 [file peerj-cs-11-2771-s001.zip › sample dataset/Test_images/Test_images/Negative/Negative_Coronary_021_RCA_Secondary7_1.png]

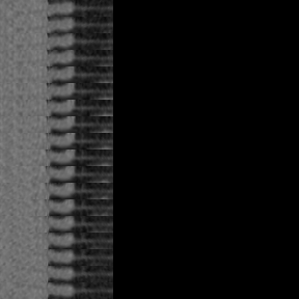

Supplement: Supplemental Information 1 [file peerj-cs-11-2771-s001.zip › sample dataset/Test_images/Test_images/Negative/Negative_Coronary_021_RCA_Secondary9_1.png]

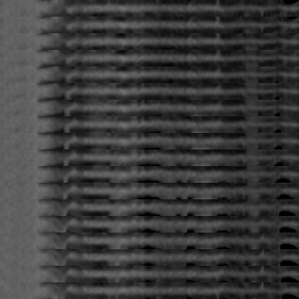

Supplement: Supplemental Information 1 [file peerj-cs-11-2771-s001.zip › sample dataset/Test_images/Test_images/Negative/Negative_Coronary_038_CX1_1.png]

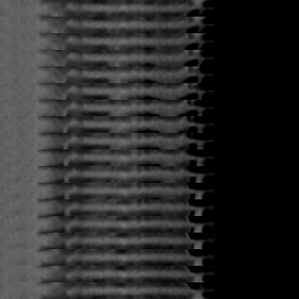

Supplement: Supplemental Information 1 [file peerj-cs-11-2771-s001.zip › sample dataset/Test_images/Test_images/Negative/Negative_Coronary_038_CX_Secondary1_1.png]

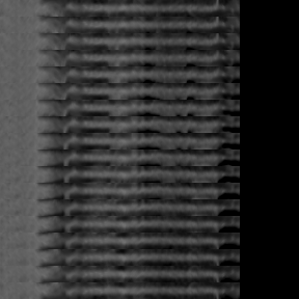

Supplement: Supplemental Information 1 [file peerj-cs-11-2771-s001.zip › sample dataset/Test_images/Test_images/Negative/Negative_Coronary_038_CX_Secondary2_1.png]

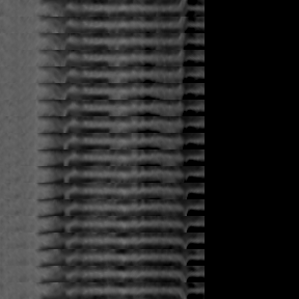

Supplement: Supplemental Information 1 [file peerj-cs-11-2771-s001.zip › sample dataset/Test_images/Test_images/Negative/Negative_Coronary_038_CX_Secondary3_1.png]

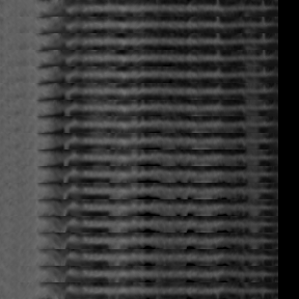

Supplement: Supplemental Information 1 [file peerj-cs-11-2771-s001.zip › sample dataset/Test_images/Test_images/Negative/Negative_Coronary_038_CX_Secondary4_1.png]

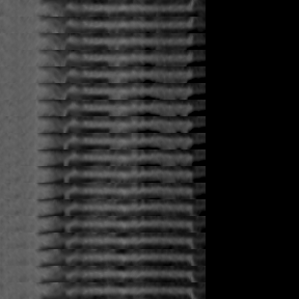

Supplement: Supplemental Information 1 [file peerj-cs-11-2771-s001.zip › sample dataset/Test_images/Test_images/Negative/Negative_Coronary_038_CX_Secondary5_1.png]

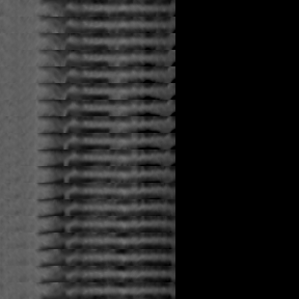

Supplement: Supplemental Information 1 [file peerj-cs-11-2771-s001.zip › sample dataset/Test_images/Test_images/Negative/Negative_Coronary_038_CX_Secondary6_1.png]

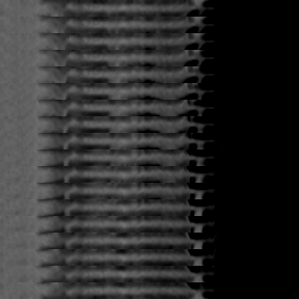

Supplement: Supplemental Information 1 [file peerj-cs-11-2771-s001.zip › sample dataset/Test_images/Test_images/Negative/Negative_Coronary_038_CX_Secondary7_1.png]

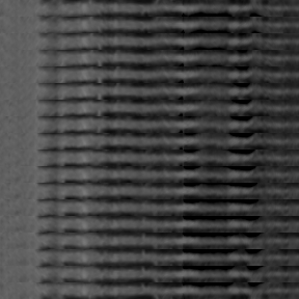

Supplement: Supplemental Information 1 [file peerj-cs-11-2771-s001.zip › sample dataset/Test_images/Test_images/Negative/Negative_Coronary_038_LAD1_1.png]

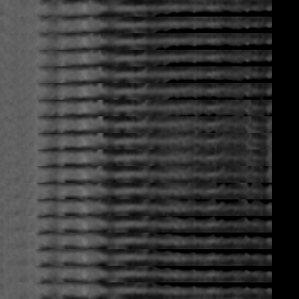

Supplement: Supplemental Information 1 [file peerj-cs-11-2771-s001.zip › sample dataset/Test_images/Test_images/Negative/Negative_Coronary_038_LAD_Secondary1_1.png]

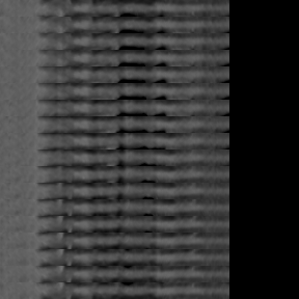

Supplement: Supplemental Information 1 [file peerj-cs-11-2771-s001.zip › sample dataset/Test_images/Test_images/Negative/Negative_Coronary_038_LAD_Secondary2_1.png]

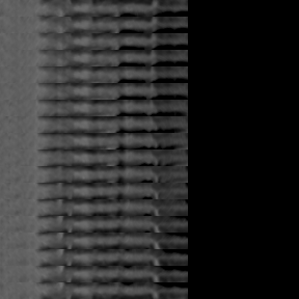

Supplement: Supplemental Information 1 [file peerj-cs-11-2771-s001.zip › sample dataset/Test_images/Test_images/Negative/Negative_Coronary_038_LAD_Secondary3_1.png]

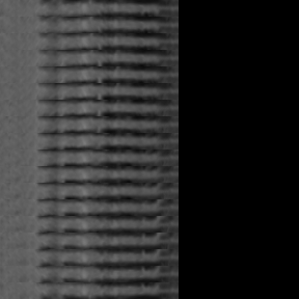

Supplement: Supplemental Information 1 [file peerj-cs-11-2771-s001.zip › sample dataset/Test_images/Test_images/Negative/Negative_Coronary_038_LAD_Secondary4_1.png]

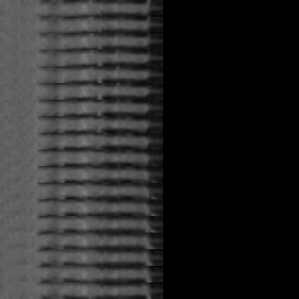

Supplement: Supplemental Information 1 [file peerj-cs-11-2771-s001.zip › sample dataset/Test_images/Test_images/Negative/Negative_Coronary_038_LAD_Secondary5_1.png]

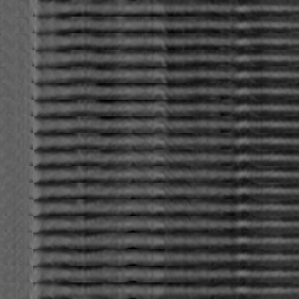

Supplement: Supplemental Information 1 [file peerj-cs-11-2771-s001.zip › sample dataset/Test_images/Test_images/Negative/Negative_Coronary_038_RCA1_1.png]

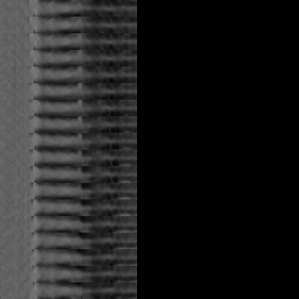

Supplement: Supplemental Information 1 [file peerj-cs-11-2771-s001.zip › sample dataset/Test_images/Test_images/Negative/Negative_Coronary_038_RCA_Secondary1_1.png]

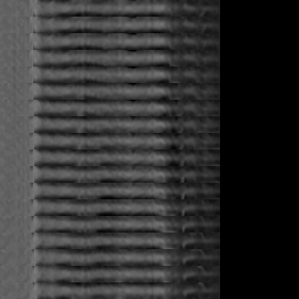

Supplement: Supplemental Information 1 [file peerj-cs-11-2771-s001.zip › sample dataset/Test_images/Test_images/Negative/Negative_Coronary_038_RCA_Secondary2_1.png]

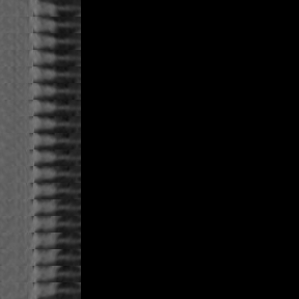

Supplement: Supplemental Information 1 [file peerj-cs-11-2771-s001.zip › sample dataset/Test_images/Test_images/Negative/Negative_Coronary_038_RCA_Secondary3_1.png]

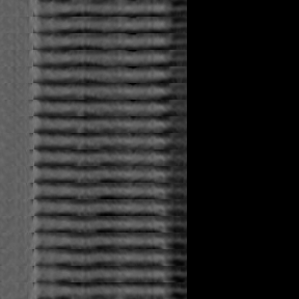

Supplement: Supplemental Information 1 [file peerj-cs-11-2771-s001.zip › sample dataset/Test_images/Test_images/Negative/Negative_Coronary_038_RCA_Secondary4_1.png]

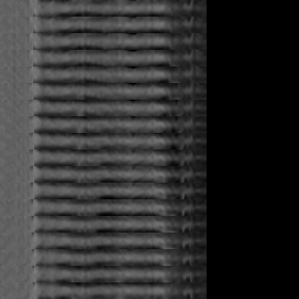

Supplement: Supplemental Information 1 [file peerj-cs-11-2771-s001.zip › sample dataset/Test_images/Test_images/Negative/Negative_Coronary_038_RCA_Secondary5_1.png]

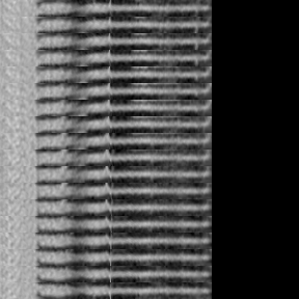

Supplement: Supplemental Information 1 [file peerj-cs-11-2771-s001.zip › sample dataset/Test_images/Test_images/Negative/Negative_Coronary_043_CX1_1.png]

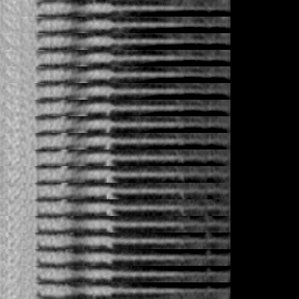

Supplement: Supplemental Information 1 [file peerj-cs-11-2771-s001.zip › sample dataset/Test_images/Test_images/Negative/Negative_Coronary_043_CX_Secondary1_1.png]

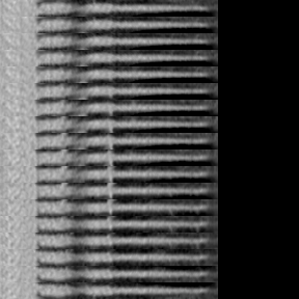

Supplement: Supplemental Information 1 [file peerj-cs-11-2771-s001.zip › sample dataset/Test_images/Test_images/Negative/Negative_Coronary_043_CX_Secondary2_1.png]

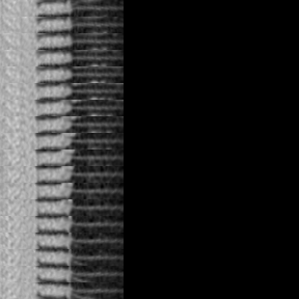

Supplement: Supplemental Information 1 [file peerj-cs-11-2771-s001.zip › sample dataset/Test_images/Test_images/Negative/Negative_Coronary_043_CX_Secondary3_1.png]

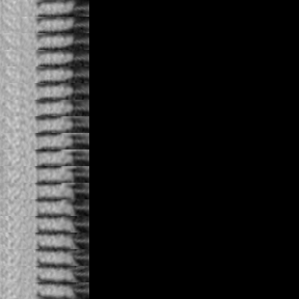

Supplement: Supplemental Information 1 [file peerj-cs-11-2771-s001.zip › sample dataset/Test_images/Test_images/Negative/Negative_Coronary_043_CX_Secondary4_1.png]

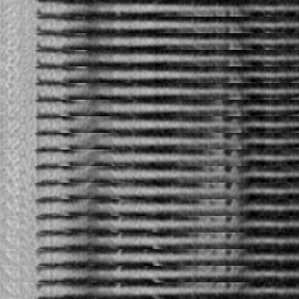

Supplement: Supplemental Information 1 [file peerj-cs-11-2771-s001.zip › sample dataset/Test_images/Test_images/Negative/Negative_Coronary_043_LAD1_1.png]

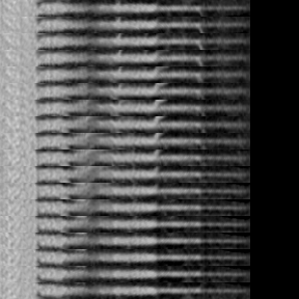

Supplement: Supplemental Information 1 [file peerj-cs-11-2771-s001.zip › sample dataset/Test_images/Test_images/Negative/Negative_Coronary_043_LAD_Secondary1_1.png]

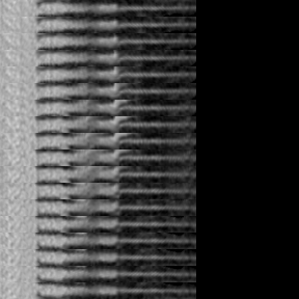

Supplement: Supplemental Information 1 [file peerj-cs-11-2771-s001.zip › sample dataset/Test_images/Test_images/Negative/Negative_Coronary_043_LAD_Secondary2_1.png]

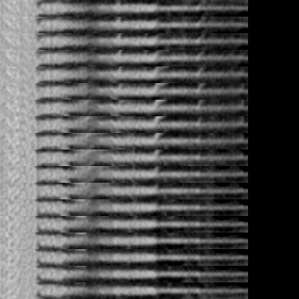

Supplement: Supplemental Information 1 [file peerj-cs-11-2771-s001.zip › sample dataset/Test_images/Test_images/Negative/Negative_Coronary_043_LAD_Secondary3_1.png]

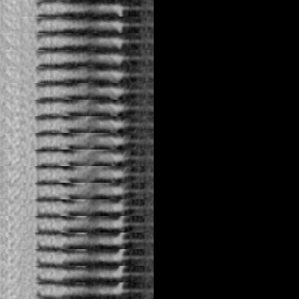

Supplement: Supplemental Information 1 [file peerj-cs-11-2771-s001.zip › sample dataset/Test_images/Test_images/Negative/Negative_Coronary_043_LAD_Secondary4_1.png]

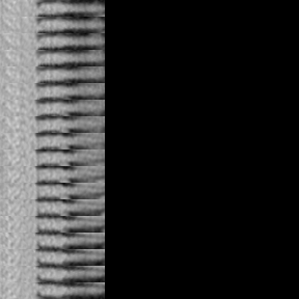

Supplement: Supplemental Information 1 [file peerj-cs-11-2771-s001.zip › sample dataset/Test_images/Test_images/Negative/Negative_Coronary_043_LAD_Secondary5_1.png]

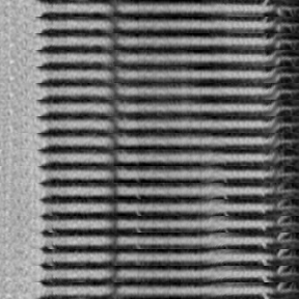

Supplement: Supplemental Information 1 [file peerj-cs-11-2771-s001.zip › sample dataset/Test_images/Test_images/Negative/Negative_Coronary_043_RCA1_1.png]

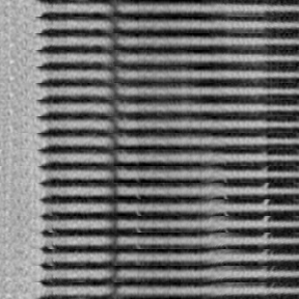

Supplement: Supplemental Information 1 [file peerj-cs-11-2771-s001.zip › sample dataset/Test_images/Test_images/Negative/Negative_Coronary_043_RCA_Secondary1_1.png]

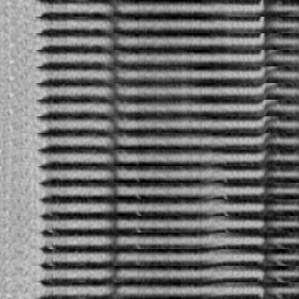

Supplement: Supplemental Information 1 [file peerj-cs-11-2771-s001.zip › sample dataset/Test_images/Test_images/Negative/Negative_Coronary_043_RCA_Secondary2_1.png]

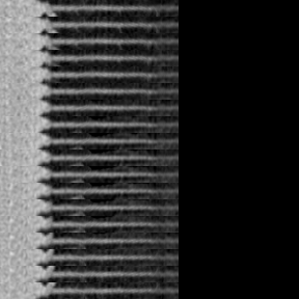

Supplement: Supplemental Information 1 [file peerj-cs-11-2771-s001.zip › sample dataset/Test_images/Test_images/Negative/Negative_Coronary_043_RCA_Secondary3_1.png]

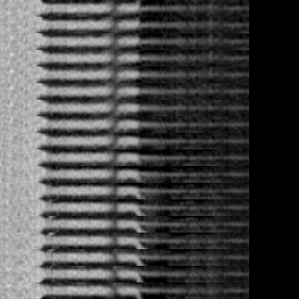

Supplement: Supplemental Information 1 [file peerj-cs-11-2771-s001.zip › sample dataset/Test_images/Test_images/Negative/Negative_Coronary_043_RCA_Secondary4_1.png]

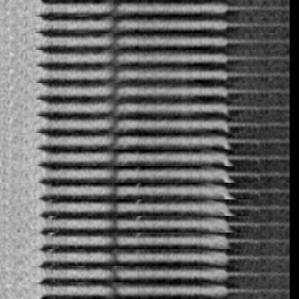

Supplement: Supplemental Information 1 [file peerj-cs-11-2771-s001.zip › sample dataset/Test_images/Test_images/Negative/Negative_Coronary_043_RCA_Secondary5_1.png]

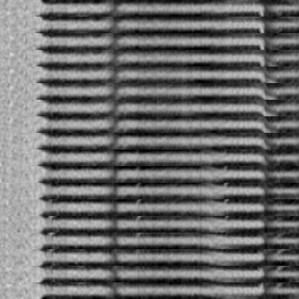

Supplement: Supplemental Information 1 [file peerj-cs-11-2771-s001.zip › sample dataset/Test_images/Test_images/Negative/Negative_Coronary_043_RCA_Secondary6_1.png]

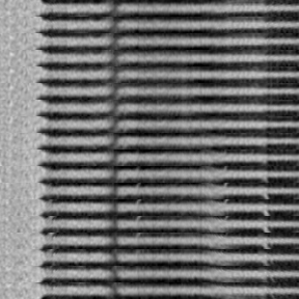

Supplement: Supplemental Information 1 [file peerj-cs-11-2771-s001.zip › sample dataset/Test_images/Test_images/Negative/Negative_Coronary_043_RCA_Secondary7_1.png]

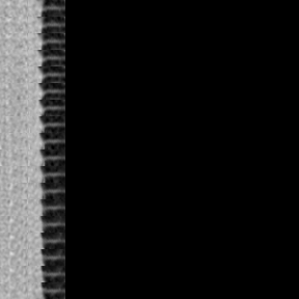

Supplement: Supplemental Information 1 [file peerj-cs-11-2771-s001.zip › sample dataset/Test_images/Test_images/Negative/Negative_Coronary_043_RCA_Secondary8_1.png]

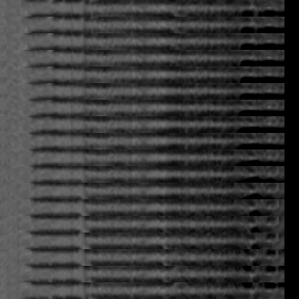

Supplement: Supplemental Information 1 [file peerj-cs-11-2771-s001.zip › sample dataset/Test_images/Test_images/Negative/Negative_Coronary_068_CX1_1.png]

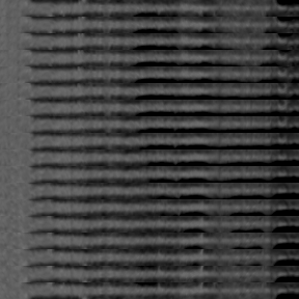

Supplement: Supplemental Information 1 [file peerj-cs-11-2771-s001.zip › sample dataset/Test_images/Test_images/Negative/Negative_Coronary_068_CX_Secondary1_1.png]

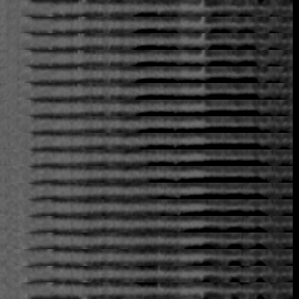

Supplement: Supplemental Information 1 [file peerj-cs-11-2771-s001.zip › sample dataset/Test_images/Test_images/Negative/Negative_Coronary_068_CX_Secondary2_1.png]

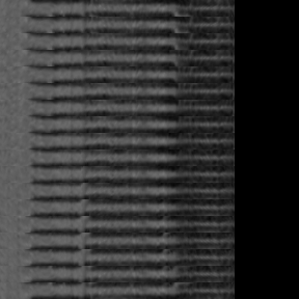

Supplement: Supplemental Information 1 [file peerj-cs-11-2771-s001.zip › sample dataset/Test_images/Test_images/Negative/Negative_Coronary_068_CX_Secondary3_1.png]

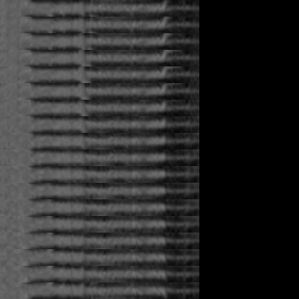

Supplement: Supplemental Information 1 [file peerj-cs-11-2771-s001.zip › sample dataset/Test_images/Test_images/Negative/Negative_Coronary_068_CX_Secondary4_1.png]

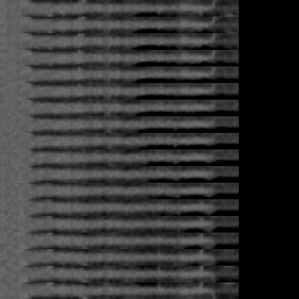

Supplement: Supplemental Information 1 [file peerj-cs-11-2771-s001.zip › sample dataset/Test_images/Test_images/Negative/Negative_Coronary_068_CX_Secondary5_1.png]
